# The Importance of Follow-Up in Adolescent Patients With a History of Ovarian Dermoid Cysts: A Case Report

**DOI:** 10.7759/cureus.81188

**Published:** 2025-03-25

**Authors:** Christina F Saint-Ilma, Tamar L Levene

**Affiliations:** 1 Surgery, Florida International University, Herbert Wertheim College of Medicine, Miami, USA; 2 Pediatric Surgery, Joe DiMaggio Children’s Hospital, Hollywood, USA

**Keywords:** anti-nmda receptor antibodies, neuropsychiatric symptom, ovarian dermoid cyst, patient follow-up, premature menopause

## Abstract

Ovarian dermoid cysts (ODCs), also known as mature cystic teratomas, are the most common ovarian neoplasms in children and adolescents. While typically asymptomatic, ODCs can present with a range of symptoms, including neuropsychiatric disturbances due to paraneoplastic encephalitis. The absence of universally accepted guidelines for post-operative surveillance poses a significant challenge in managing these cases, particularly in adolescent patients.

We report the case of a previously healthy 12-year-old girl who initially presented with altered mental status and psychosis, leading to the discovery of bilateral ODCs. Despite successful laparoscopic ovarian-sparing resections of the initial lesions, as well as a laparoscopic ovarian-sparing resection of a unilateral recurrence one year later, the patient was lost to follow-up and did not undergo routine surveillance. She eventually presented several years later with severe abdominal distention and frequent seizures, which an abdominal ultrasound revealed were due to recurrent bilateral ODCs that encompassed the entirety of the abdomen. Upon evaluation with MRI, no normal ovarian tissue was identified. After discussion with the patient and her mother, she ultimately underwent a bilateral salpingo-oophorectomy at age 20, resulting in premature menopause.

This case underscores the necessity of standardized follow-up protocols for adolescents with ODCs that prioritize regular imaging and clinical monitoring to identify recurrent lesions and prevent adverse outcomes. Additionally, it emphasizes the importance of multidisciplinary care and patient education to ensure adherence to follow-up recommendations. Our findings suggest that a more consistent approach to post-operative surveillance could prevent severe complications and improve long-term outcomes for adolescent patients with ODCs.

## Introduction

Ovarian dermoid cysts (ODCs), or mature cystic teratomas, are the most common ovarian neoplasms in children and adolescents [1]. These masses, a type of germ cell tumor composed of totipotent cells, have the potential to differentiate into any cell type, including embryonic and extraembryonic tissue. ODCs are usually unilateral, although bilateral presentation has been noted in approximately 10-20% of cases [1,2]. While ODCs are typically asymptomatic, symptomatic patients may present with abdominal pain and, less commonly, a palpable mass, nausea, vomiting, menstrual abnormalities, precocious puberty, and virilization [3]. Rarely, they can cause encephalitis, altered mental status, headaches, and seizures due to paraneoplastic encephalitis secondary to N-methyl-D-aspartate (NMDA) receptor antibodies being secreted from the ODC [4]. In a retrospective cohort study that reviewed 233 patients who had surgical resection of ODCs, less than 1 percent (0.85%) were diagnosed with anti-NMDA receptor encephalitis [5]. As severe dysfunction may result from these cases, prompt diagnosis followed by surgical removal of the antibody-secreting masses is imperative, combined with continued follow-up to promote a good prognosis.

Our case highlights this rare presentation of ODCs in an adolescent patient with multiple recurrences, subsequently developing and ultimately necessitating bilateral salpingo-oophorectomy. Although there is not a universally accepted guideline for post-operative surveillance following ODC removal, our case, along with a review of the literature, highlights the importance of ongoing surveillance and patient follow-up, particularly in adolescent patients. This manuscript was prepared following the CARE guidelines (https://www.care-statement.org).

## Case presentation

**Figure 1:**
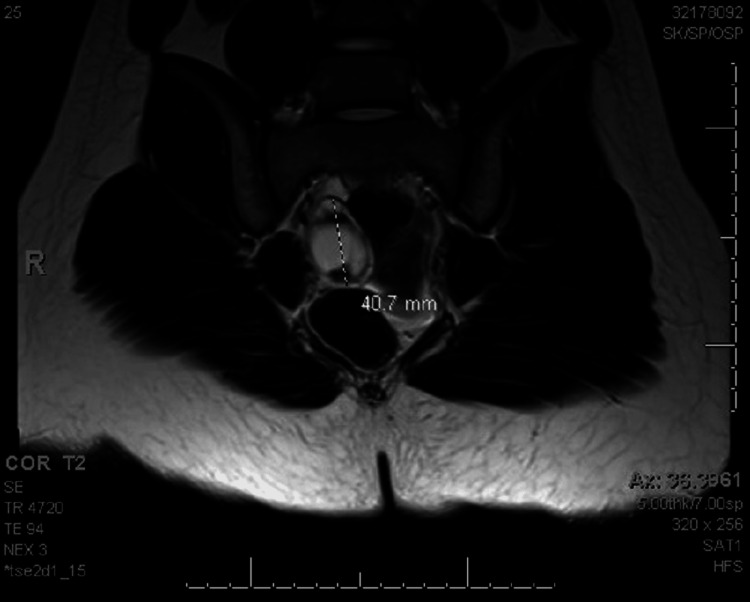
MRI of the abdomen with and without contrast demonstrating one of the masses discovered on initial presentation in 2016.

**Figure 2:**
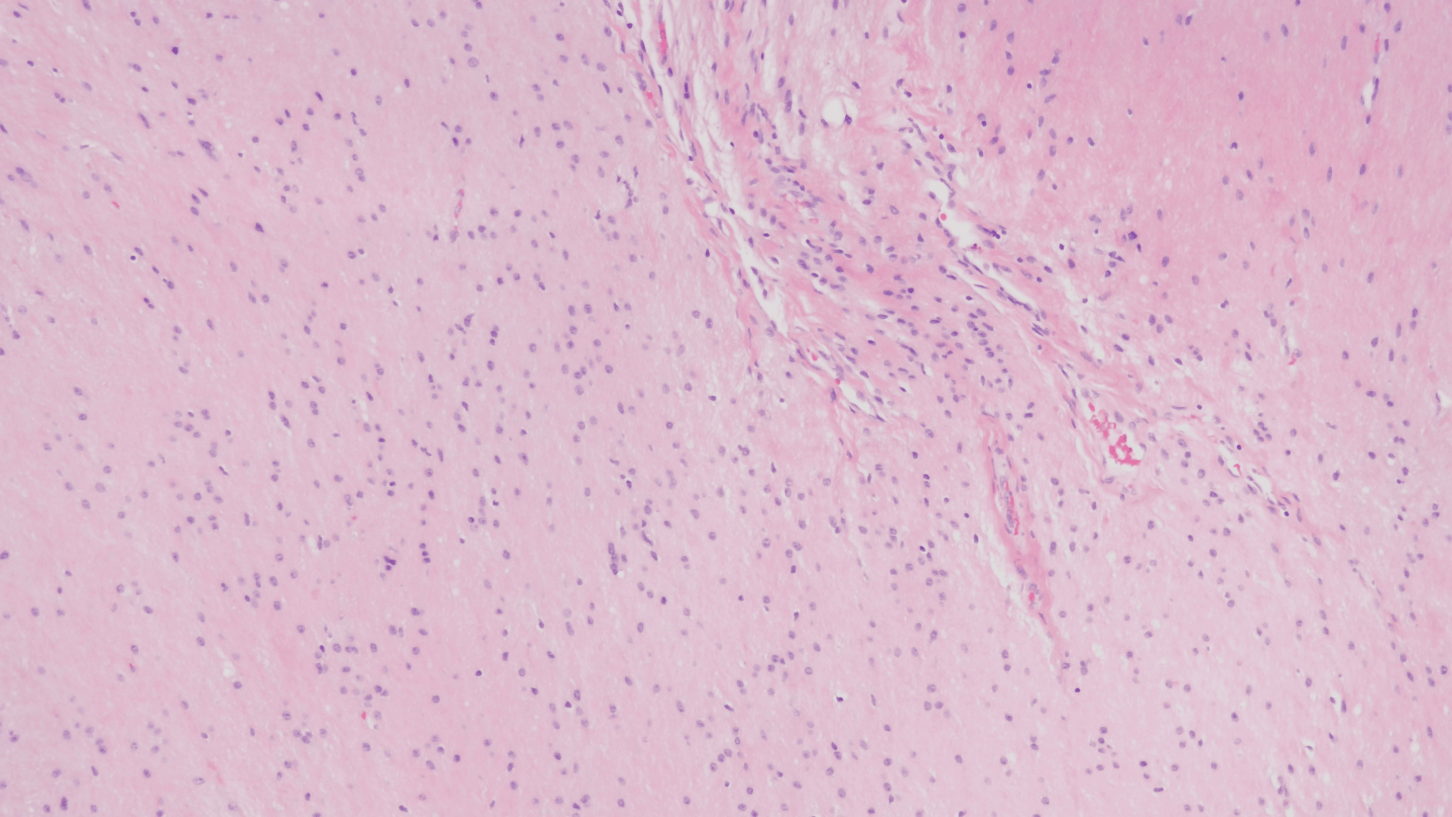
Pathology demonstrating mature neuroglial tissue from the ODCs removed in 2016. ODC: Ovarian dermoid cyst.

A previously healthy 12-year-old girl presented to the ED with a two-day history of altered mental status, perseveration, and auditory and visual hallucinations. Notably, the patient had started her first menstrual period five days prior, and her mother had brought her in due to complaints of painful menstrual cramps along with an acute change in persona. There was no recent trauma, illness, or overt seizure activity. The patient denied any recent abuse, ingestions or drug use, difficulty at school, or problems at home. Physical exam findings showed evidence of severe cognitive impairment, agitation, and a lack of orientation to place or time. Basic laboratory and toxicology studies were unremarkable. MRI revealed bilateral ovarian masses with calcifications and adipose tissue concerning for ODCs (Figure 1). Based on these findings, the patient underwent laparoscopic resection of bilateral ovarian masses at which time a left ovarian teratoma and paratubal cyst were excised along with a right-sided ovarian fatty mass consistent with a teratoma. Final pathology revealed no evidence of malignancy with bilateral mature cystic teratomas with neuroglial tissue (Figure 2). The patient tolerated the procedure well with no operative complications and subsequently received intravenous immunoglobulin (IVIG) as recommended by a pediatric immunologist and was discharged to home on post-operative day (POD) 20 in good condition. The patient was advised to return for ongoing surveillance imaging every three months following surgery or sooner if her symptoms recurred.

One year post-op, the patient presented with a recurrence of a mass in her right ovary which was subsequently resected in its entirety laparoscopically. There was no evidence of any identifiable cysts or lesions on the left ovary, and the patient was discharged home in good condition on POD one. Pathology results confirmed the presence of a mature cystic teratoma. Following this surgery, a pelvic ultrasound was performed that demonstrated normal adnexa bilaterally without any masses or cysts. As such, recommendations were made once again for ongoing surveillance with ultrasound every three months; however, the patient was lost to follow-up.

**Figure 3:**
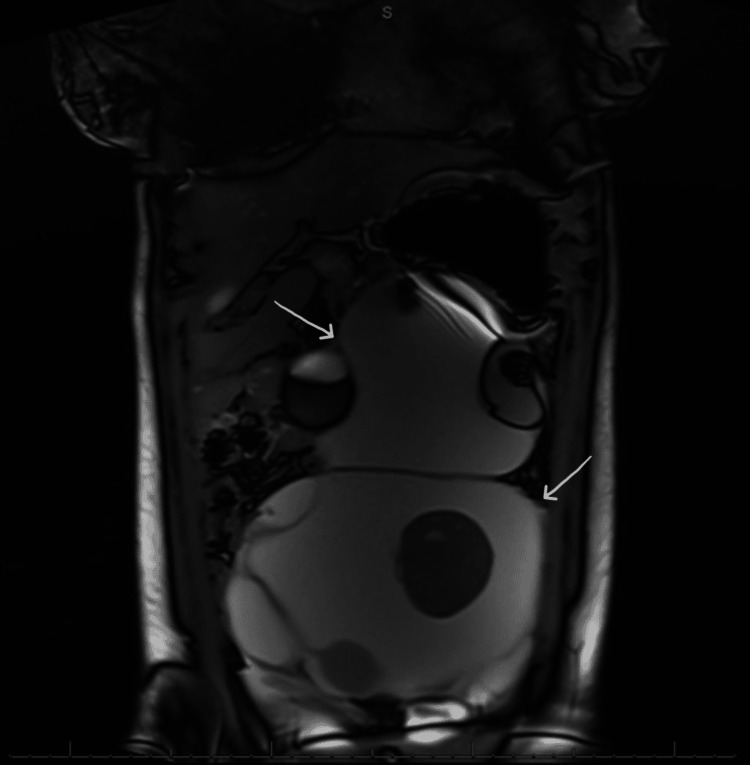
MRI of the abdomen with and without contrast demonstrating large central multilobed cystic structures with solid components, consistent with ovarian dermoid cysts (white arrows).

Seven years following her initial presentation, the patient sought out care again due to experiencing some lower abdominal fullness; at that time, she was noted to have a 32 cm complex mass extending from the pelvis to the upper abdomen appreciated on ultrasound and MRI (Figure 3), that was concerning for a recurrence. Notably, no normal ovarian stroma was appreciated on imaging. Upon further questioning, the patient’s mother admitted that the patient had been having seizures that were becoming more frequent; she had attributed this to a lack of compliance with medical therapy. Given these findings, the patient underwent exploratory laparotomy with bilateral salpingo-oophorectomy as the large teratomas were noted from each ovary with complete obliteration of normal ovarian tissue. The pathology report confirmed the presence of 19.4 cm and 22.0 cm left and right ODCs, respectively. The most recent follow-up was in April 2024, where the patient reported she was doing well without any further neurological symptoms. Menopausal symptoms were noted; however, she reported plans to seek care from a gynecologist and endocrinologist for further management and recommendations.

## Discussion

The management of ODCs in the adolescent population involves a balance between the complete removal of all neoplastic tissue and the preservation of future fertility. Early detection of ODCs, along with appropriate surveillance, is vital for maintaining normal ovarian tissue. The follow-up interval for ODCs post-surgical resection varies based on physician practice due to the lack of a universal guideline. In this case report, we highlight an adolescent girl with recurrent ODCs who was eventually lost to follow-up, leading to the growth of large, bilateral ODCs that required removal and the performance of a bilateral salpingo-oophorectomy. As a result, she now faces the challenges of menopause at just 20 years old, an outcome that might have been prevented with appropriate and consistent post-operative surveillance. We have reviewed the literature to further guide management and surveillance recommendations for future patients.

As previously mentioned, patients with ODCs may or may not have symptoms, and symptomatic presentations can vary greatly from abdominal pain to psychosis, as seen in our patient. The link between ODCs and neuropsychiatric disorders, particularly anti-NMDA receptor encephalitis, is well-documented but often underrecognized. Early identification and treatment are vital, as studies have shown that up to 80% of patients with anti-NMDA encephalitis improve with early immunotherapy or tumor removal [5,6]. In our case, the patient’s psychosis, which initially masked the underlying condition, was a critical diagnostic clue that led to the discovery of her bilateral ODCs. This case illustrates the importance of a multidisciplinary approach in managing such patients, involving psychiatry, neurology, gynecology, and pediatric surgery to ensure comprehensive care. In cases of new-onset psychosis, a multidisciplinary approach should be taken to facilitate early recognition, which is important for a good prognosis [6].

In cases of recurrence, surgical intervention is predominantly based on the size and growth rate of the tumor. The use of a laparoscopic technique for dermoid cyst removal is preferred; however, in cases where the mass is larger than 8 cm, a laparotomy is ideal to avoid complications [1]. Surgery may be warranted in masses that are greater than or equal to 5 cm, particularly in adolescent patients, based on the increased risk for loss of fertility [1,7]. In patients experiencing psychosis secondary to ODC, surgical removal is warranted regardless of the size of the mass due to the threat of permanent neurological damage [4].

Currently, there is evidence to support annual follow-up with imaging and pelvic exams in adolescents. However, some clinicians have argued against continued surveillance based on a low chance of recurrence, the slow growth rate of the tumor, and a low chance of malignancy [3]. In a retrospective cohort study, 66 patients with ODCs who had undergone either laparotomy or laparoscopic tumor removal were analyzed, and a surveillance protocol was proposed [7]. The protocol entailed that all adolescent patients receive a maximum 12-month follow-up post-op with ultrasound. If patients have a negative ultrasound at 12 months post-op, they can follow up as needed if anything changes. However, if recurrence is detected, an earlier three-to-six-month ultrasound surveillance period should be followed, and surgical intervention should be considered for large tumors [7].

That same study found that 11% of those patients had recurrent or persistent cysts, compared to a 4-6% recurrence rate in adult patients [7]. Based on the increased risk in this population, we recommend the adoption of a more standardized follow-up protocol for adolescents with ODCs that emphasizes regular ultrasound imaging and close monitoring, particularly in the first year post-resection. Multidisciplinary care is essential in managing complex cases with neuropsychiatric involvement and also in promoting follow-up through coordination between primary care providers and specialists. Patient and family education on the risks of recurrence and the necessity of consistent follow-up is crucial in preventing adverse outcomes that can occur due to large tumor size, such as loss of fertility.

## Conclusions

Our case underscores the importance of vigilant follow-up in adolescent patients with ODCs, particularly those who present with atypical symptoms such as neuropsychiatric disturbances. Our patient's progression from initial presentation with psychosis to the eventual development of recurrent large, bilateral ODCs necessitating bilateral salpingo-oophorectomy highlights the severe consequences of loss to follow-up. This outcome, resulting in premature menopause at the age of 20, likely could have been avoided with consistent post-operative surveillance.

Although there are likely many factors that contributed to the lack of follow-up in this case, the variability in post-operative follow-up practices presents a significant challenge in managing ODCs. Our case illustrates the potential risks associated with this variability, emphasizing the need for a more standardized approach. Regular imaging and close clinical monitoring, especially in the critical first year following surgical intervention, should be prioritized to detect recurrences early and to preserve ovarian function. Additionally, this case also highlights the rare incidence of ODCs and neuropsychiatric symptoms, such as those caused by anti-NMDA receptor antibody encephalitis. Early recognition and management in these cases are crucial, not only for addressing the underlying gynecological pathology but also to prevent any long-term neurological damage. This case should serve as a compelling reminder to clinicians of the need for ongoing surveillance and comprehensive care in the management of adolescent patients with a history of ODCs.
